# Metabolomic Profiling of Pulmonary Neuroendocrine Neoplasms

**DOI:** 10.3390/cancers16183179

**Published:** 2024-09-17

**Authors:** Clémence Boullier, Fabien C. Lamaze, Jean-François Haince, Rashid Ahmed Bux, Michèle Orain, Jiamin Zheng, Lun Zhang, David S. Wishart, Yohan Bossé, Venkata S. K. Manem, Philippe Joubert

**Affiliations:** 1 Centre de Recherche de l’institut Universitaire de Cardiologie et de Pneumologie de Québec (IUCPQ), Quebec City, QC G1V 4G5, Canada; clemence.boullier.1@ulaval.ca (C.B.); fabien.lamaze@criucpq.ulaval.ca (F.C.L.); michele.orain@criucpq.ulaval.ca (M.O.); yohan.bosse@criucpq.ulaval.ca (Y.B.); 2 Faculty of Medicine, Laval University, Quebec City, QC G1V 0A6, Canada; venkata.manem@crchudequebec.ulaval.ca; 3 BioMark Diagnostics Inc., Richmond, BC V6X 2W8, Canada; jhaince@biomarkdiagnostics.com (J.-F.H.); rahmed@biomarkdiagnostics.com (R.A.B.); 4 The Metabolomics Innovation Center (TMIC), University of Alberta, Edmonton, AB T6G 1C9, Canada; jiamin3@ualberta.ca (J.Z.); lun2@ualberta.ca (L.Z.); dwishart@ualberta.ca (D.S.W.); 5 Department of Mathematics and Computer Science, University of Quebec at Trois-Riviere, Trois-Riviere, QC G8Z 4M3, Canada; 6 Centre de Recherche du CHU de Québec, Quebec City, QC G1E 6W2, Canada

**Keywords:** neuroendocrine neoplasm (NEN), lung, metabolomic profile, metabolism, carcinoid tumors, small-cell lung carcinoma (SCLC), large-cell neuroendocrine carcinoma (LCNEC), blood metabolites

## Abstract

**Simple Summary:**

Pulmonary neuroendocrine neoplasms (NENs) are a challenging type of lung cancer due to their varied clinical features and aggressive behavior. This study aimed to find specific metabolomic profiles in the blood of patients with different subtypes of lung NENs, which could help in early diagnosis. By analyzing 153 metabolites in the plasma of 120 NEN patients and comparing them with healthy individuals and patients with other lung cancers, we identified specific metabolic changes. These findings could lead to new biomarkers for early detection and better management of lung NENs, ultimately improving patient outcomes.

**Abstract:**

Background/Objectives: Pulmonary neuroendocrine neoplasms (NENs) account for 20% of malignant lung tumors. Their management is challenging due to their diverse clinical features and aggressive nature. Currently, metabolomics offers a range of potential cancer biomarkers for diagnosis, monitoring tumor progression, and assessing therapeutic response. However, a specific metabolomic profile for early diagnosis of lung NENs has yet to be identified. This study aims to identify specific metabolomic profiles that can serve as biomarkers for early diagnosis of lung NENs. Methods: We measured 153 metabolites using liquid chromatography combined with mass spectrometry (LC-MS) in the plasma of 120 NEN patients and compared them with those of 71 healthy individuals. Additionally, we compared these profiles with those of 466 patients with non-small-cell lung cancers (NSCLCs) to ensure clinical relevance. Results: We identified 21 metabolites with consistently altered plasma concentrations in NENs. Compared to healthy controls, 18 metabolites were specific to carcinoid tumors, 5 to small-cell lung carcinomas (SCLCs), and 10 to large-cell neuroendocrine carcinomas (LCNECs). These findings revealed alterations in various metabolic pathways, such as fatty acid biosynthesis and beta-oxidation, the Warburg effect, and the citric acid cycle. Conclusions: Our study identified biomarker metabolites in the plasma of patients with each subtype of lung NENs and demonstrated significant alterations in several metabolic pathways. These metabolomic profiles could potentially serve as biomarkers for early diagnosis and better management of lung NENs.

## 1. Introduction

Lung cancer is the leading cause of cancer deaths, representing a major public health issue worldwide [1,2]. Among the new diagnosed lung cancers, 20% originate from the pulmonary neuroendocrine system [3]. Pulmonary neuroendocrine neoplasms (NENs) include carcinoid tumors, small-cell carcinomas (SCLCs), and large-cell neuroendocrine carcinomas (LCNECs) [2]. Carcinoid tumors are the least aggressive of the NENs [4], with a 10-year survival rate of 58–83% [5]. In contrast, neuroendocrine carcinomas (NECs), which include SCLCs and LCNECs, are aggressive tumors with a 17% survival rate of 10 years [6]. NECs are generally diagnosed at an advanced stage, which limits the therapeutic options [7,8]. Currently, the diagnosis of NENs relies on histopathology or cytology evaluation and requires access to the tumor. Over the last decade, liquid biopsy (blood or other body fluids) has gained interest as a surrogate approach to diagnose pulmonary tumors. The advantages of a liquid biopsy are numerous: easy access to blood, quick turnaround time, and the possibility of making a diagnosis when a tissue biopsy is not feasible.

Metabolomics is an expanding field of study that could provide clinicians easier access to NEN biomarkers in biofluids. Since Warburg’s seminal work in the 1920s on the aerobic glycolysis metabolism in cancer [9], many dysregulated metabolisms were described, such as the increase in glutamine or anabolic fatty acids [10]. It is now accepted that the metabolism of cancer cells differ from that of normal cells under homeostatic physiological conditions, promoting tumorigenesis and the development of drug resistance [10]. Indeed, metabolic dysfunction was included as a hallmark of cancer in 2011 [11]. The association between increased or decreased concentrations of blood metabolites and cancer suggests that the deregulated metabolism of cancer cells is reflected by the altered presence of metabolites in the blood [12]. While metabolomic profiles are available for some cancers [13,14], there is still a lack of information about metabolic dysregulation in NENs, particularly for LCNECs.

In the present study, we hypothesize that tumor cells or cells in the surrounding tumor microenvironment directly or indirectly impact the patients’ plasma metabolite composition. As such, we evaluated whether the plasma metabolic composition of NEN patients was distinctive from that of healthy controls. We further identified metabolites specific to carcinoid tumors, SCLCs, and LCNECs. Finally, we assessed the specificity of potential NEN biomarkers in comparison to 466 non-small-cell lung cancer (NSCLC) cases.

## 2. Materials and Methods

### 2.1. Study Population

This is a case–control study comprising 657 consecutive participants who visited the Institut de cardiologie et de pneumologie de Québec-Université Laval (IUCPQ-UL) between 2005 and 2021. A consent form was obtained for all participants by the IUCPQ biobank. The cohort included 120 patients with NENs and 466 with NSCLCs, all of whom had lung resections, as well as 71 healthy individuals without pulmonary pathology. Plasma was collected from these individuals on the day of the surgery for the patients with cancer, prior to the resection. Plasma samples were stored at −70 °C. The protocol was approved by the Research Ethics Committee of the IUCPQ (2022-3781, 22164). Frozen plasma samples of 200 or 400 µL were shipped to the Metabolomic Innovation Centre (TMIC), at the University of Alberta, Canada, for quantitative metabolomic analysis.

### 2.2. Metabolomic Profiling

A fully quantitative targeted mass spectrometry (MS) analysis, targeting 166 metabolites, was performed on all samples. Metabolomic assays utilized high-performance liquid chromatography (HPLC) named Agilent 1100 HPLC (Agilent Technologies, Santa Clara, CA, USA) in combination with an AB Sciex 4000 QTrap^®^ tandem mass (MS/MS) spectrometer (Applied Biosystems/MDS Analytical Technologies, Foster City, CA, USA) and were constructed as described previously [15]. Plasma samples were thawed on ice, vortexed, and centrifuged at 18,000× *g*. A 10 µL aliquot of each sample was loaded onto a filter insert in a 96-well plate and dried under nitrogen. Metabolites were derivatized using phenylisothiocyanate (PITC) and extracted with methanol containing 5 mM ammonium acetate. The extracts were obtained by centrifugation of the double plate system, allowing for the targeted identification and quantification of metabolites. For amino acids, biogenic amines, carnitines, and lipids, the extracts were diluted appropriately before mass spectrometric analysis. Organic acids were analyzed by mixing 50 µL of plasma with an internal standard mixture and ice-cold methanol, followed by overnight protein precipitation at −20 °C. After centrifugation, derivatization with 3-nitrophenylhydrazine (3-NPH) and other reagents was performed before injection into the HPLC-MS/MS system. The assay integrated isotope-labeled internal standards for accurate quantification, covering a wide range of metabolites, including amino acids, organic acids, biogenic amines, acylcarnitines, glycerophospholipids, sphingolipids, and sugars.

Metabolites with more than 50% of missing values were excluded from further analysis, leaving 153 metabolites. Metabolite levels were standardized for analysis using z-scores. To quantify the amplitude of the observed changes in metabolite levels between the control and cancer patient groups, we used fold change (FC). The calculation formula used was FC = Y/X, where X represents the value of the metabolic concentration in the healthy controls or in the NSCLC control patients, and Y represents the value of the metabolic concentration in NEN patients.

### 2.3. Statistical Analyses

All statistical analyses were performed using the RStudio desktop application with R version 4.2.1 (Posit Software PBC, Boston, MA, USA). The significance threshold for statistical tests was set at 5%. We tested for differences in age, sex, smoking status, and BMI between groups of NEN patients, healthy controls, and NSCLC patients. A Student’s *t*-test was used to compare ages and BMIs, and a chi-square test was used to compare the distribution of sex and smoking status. Concentration data were normalized by z-scores. For the first step of metabolite selection, a bootstrap resampling strategy was carried out 10,000 times to mitigate unknown associations or unwanted batch effects, with a sampling size equal to the smallest group (*n* = x). For each bootstrap iteration, a Mann–Whitney test was performed on the resampled populations to compare metabolite concentrations between groups of interest. The Mann–Whitney statistic and *p*-value were recorded, and a metabolite was deemed impacted if the median *p*-value passed a Bonferroni correction at a threshold *p*-value of 0.0003 (0.05/153). As a second step of metabolite selection, we used a backward elimination approach using regularization regression models (lasso, ridge, or elastic net) using the train function in the glmnet package. For internal cross validation of the model and to control for overfitting, we used a *k*-fold cross-validation approach with 5 folds and 10 iterations. Model weights were added to balance the sample sizes and ensure a 1:1 ratio between groups. A grid search strategy was applied to identify the best combinations of α and λ hyperparameters, with α values set at 0, 0.5, or 1, and λ values set at 0.001, 0.01, 0.1, 1, or 10. For each group comparison, the best model was selected based on the highest accuracy, whether lasso (α = 1), ridge (α = 0), or elastic net (α = 0.5) regression. Model performance metrics were based on cross-validation (95% CI) and the full subset of the training group corresponding to the model. The test group was the entire cohort; given the smallest cohort size of LCNEC patients (*n* = 40), an 80/20 split would not have yielded a sufficiently large test set (*n* = 8). Pathway enrichment analysis was performed to identify associated metabolic pathways using MetaboAnalyst 6.0 available online: http://www.metaboanalyst.ca (accessed on 6 March 2024). This metabolite set enrichment analysis (MSEA) utilized the SMPDB library available online: http://www.smpdb.ca (accessed on 6 March 2024), which consists of 99 metabolite sets based on normal human metabolic pathways. The globaltest algorithm enabled the MSEA to use a generalized linear model to calculate a “Q-stat” for all metabolites [16].

### 2.4. Data Presentation

All graphical presentations were generated using an R Studio desktop application (version 4.3.1, RStudio, Boston, MA, USA). The volcano plots were created using the ggplot2 package (version 3.4.0). The metabolic pathway representation graphs were generated by MetaboAnalyst 6.0.

## 3. Results

### 3.1. Demographic and Clinical Characteristics

The demographic and clinical characteristics of NEN patients and healthy controls are presented in Table 1. NEN cases had an average of 62 years, while controls had an average age of 57 years, with no significant difference (*p* = 0.16). However, SCLC (*p* = 4.73 × 10^−2^) and LCNEC (*p* = 0.03) patients were older than controls. Sex distribution did not differ significantly between the NEN cases and controls (*p* = 0.29). Regarding smoking status, 18% of NEN cases were non-smokers, compared to 56% of controls. Due to the higher prevalence of NSCLCs in clinics, we used a cohort of NSCLC patients as a cancer outgroup to compare with NENs. The characteristics of patients with NENs were also compared with those with NSCLC patients, as shown in Table 2. As expected, patients with NSCLCs were older (65 versus 62 years old) than patients with NENs (*p* = 6.50 × 10^−4^). The distribution of smoking status was also significantly different (*p* = 1.80 × 10^−2^), but in both groups, most cases were ex-smokers (58.3% of NENs; 72.2% of NSCLCs).

### 3.2. NENs Have a Distinct Plasmatic Profile

**Figure 1:**
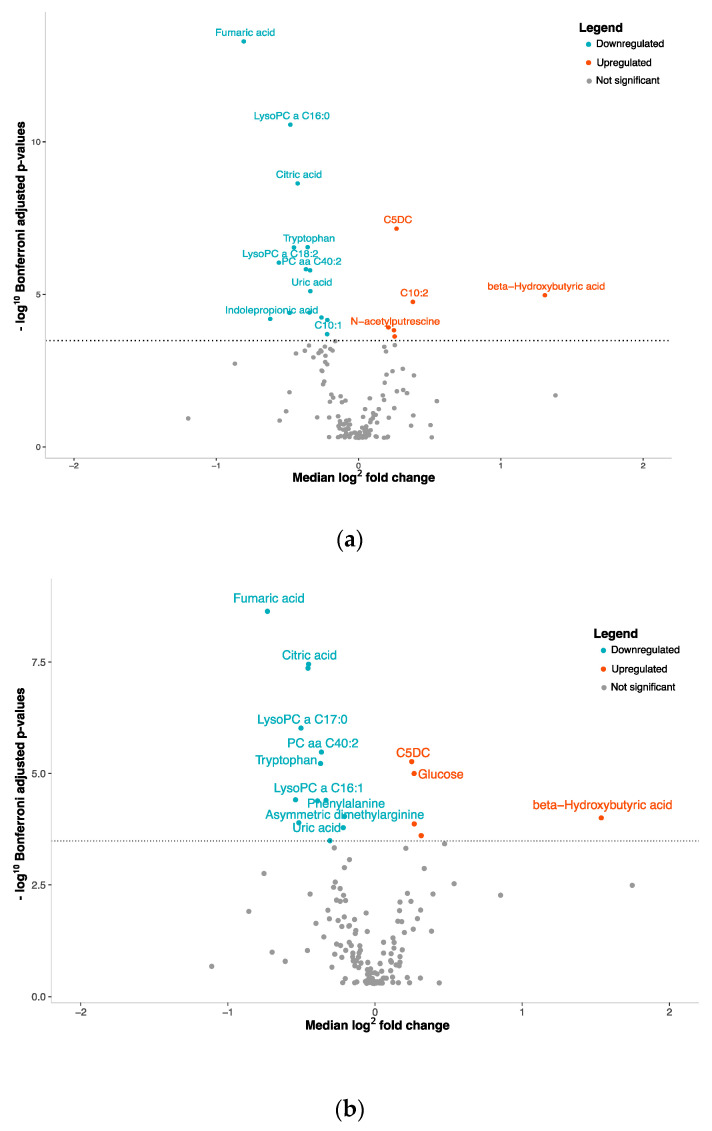

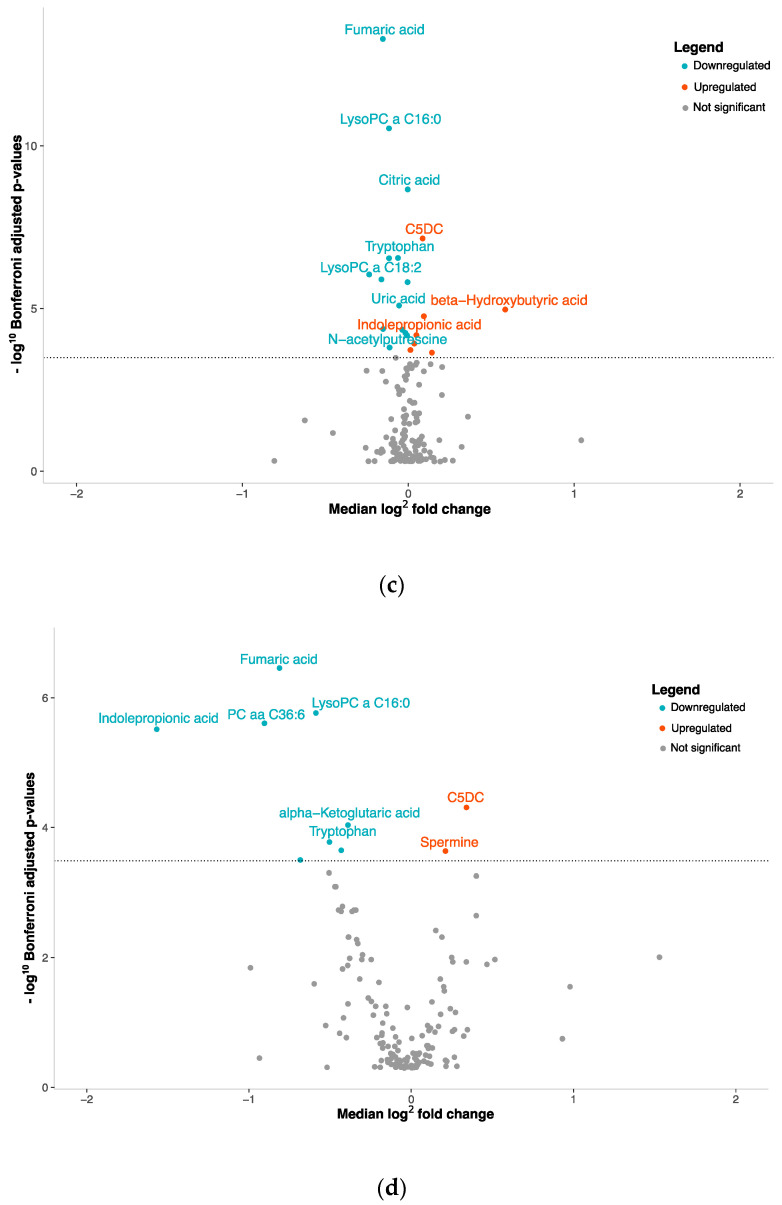

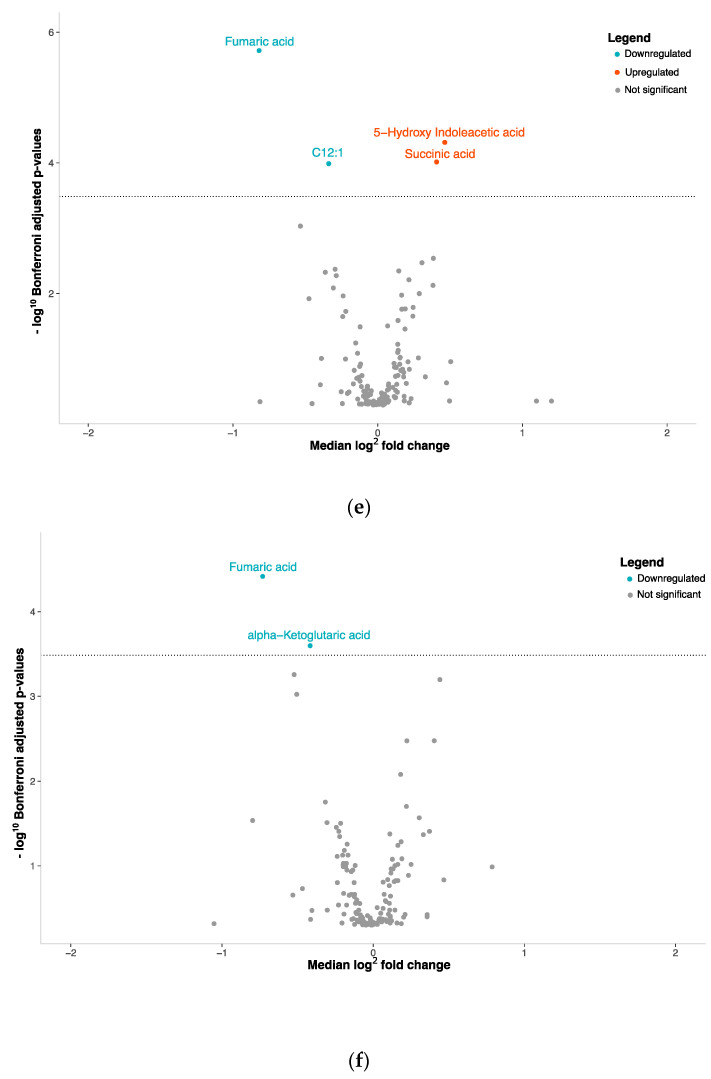
Volcano plots with plasma metabolite concentrations. Metabolite selection based on Mann–Whitney test and Bonferroni correction (*p*-value threshold = 0.0003); overexpressed metabolites = red, underexpressed = blue: (**a**) NENs grouped together, (**b**) carcinoids tumors, (**c**) SCLCs or (**d**) LCNECs compared with healthy controls. (**e**) SCLCs or (**f**) LCNECs compared to NSCLCs.

To capture the differences in metabolite concentration between NEN patients and the control group, we analyzed the plasma concentrations of 153 metabolites (Figure 1). We found that 15 metabolites displayed a significant reduction in the plasma of NEN patients, as observed with fumaric acid (*p* = 5.46 × 10^−14^) (Figure 1a, Supplementary Table S1). Conversely, six metabolites, including beta-hydroxybutyric acid (*p* = 1.02 × 10^−5^), exhibited a significant increase in concentration in NEN patients (Figure 1a, Supplementary Table S1).

First, our analysis revealed that 18 metabolites differentiated carcinoid tumors from healthy individuals, with 13 metabolites showing a significant reduction and 5 showing a significant increase in concentration (Figure 1b, Supplementary Table S2). Second, we identified four metabolites with reduced concentrations and one with an increased concentration associated with SCLCs (Figure 1c, Supplementary Table S3). Lastly, eight metabolites showed decreased plasma concentrations and two showed increased concentrations in LCNEC patients (Figure 1d, Supplementary Table S4).

To gain a deeper understanding of the metabolic distinctions within the spectrum of lung cancers, we conducted a comparative analysis between SCLCs and LCNECs against NSCLCs, which encompass both adenocarcinomas and squamous cell carcinomas. When we compared SCLCs to NSCLCs, we found significant alterations in the plasma concentrations of four metabolites (Figure 1e, Supplementary Table S5). Among these, two were increased while two were decreased. For LCNECs, we observed that only two metabolites had decreased plasma concentrations compared to NSCLC patients (Figure 1f, Supplementary Table S6).

**Figure 2:**
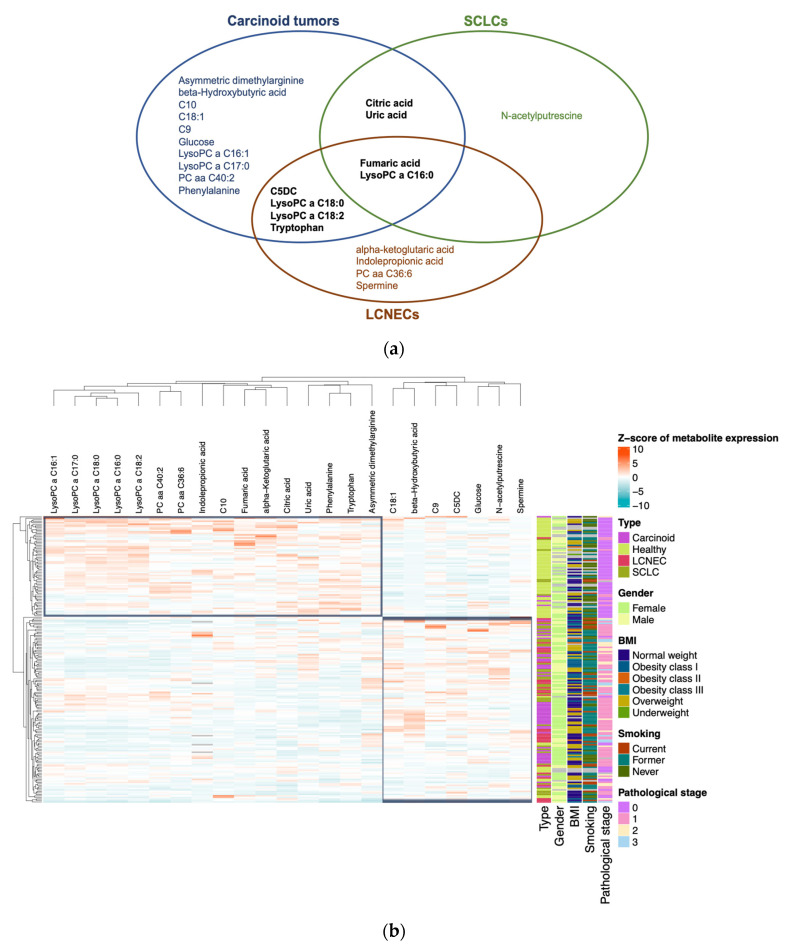
Representation of variations in concentrations of metabolites differentially expressed in plasma under different conditions and characterization of their discriminative capacity: (**a**) Venn diagram of the 23 metabolites that have significantly different concentrations between patients with carcinoid tumors, SCLCs, and LCNECs compared to healthy individuals. (**b**) Heatmap plot with plasma concentrations of these 23 metabolites. Concentrations were normalized by z-score. Clinical characteristics are integrated: gender, BMI, smoking, and pathological stage.

Further dissecting the NEN cohort, we identified 23 metabolites with altered plasma concentrations (*p* < 0.0003) in one or more NEN subtypes (carcinoid tumors, SCLC, and LCNEC) compared to healthy controls (Figure 2). Using a Venn diagram, fumaric acid and LysoPC lipid C16:0 appeared significantly in all three subtypes, while four metabolites were shared between carcinoid tumors and LCNECs, and two metabolites were common between carcinoid tumors and SCLCs (Figure 2a). Unsupervised clustering analysis revealed the segregation of healthy individuals from NEN patients, with substructuring among the NEN subtypes that was independent of clinicopathological information (Figure 2b). Specifically, we identified upregulated glucose (in carcinoid tumors), N-acetylputrescine (in SCLC), and spermine (in LCNEC) as metabolites uniquely associated with specific NEN subtypes.

### 3.3. Pathway Analysis

**Figure 3:**
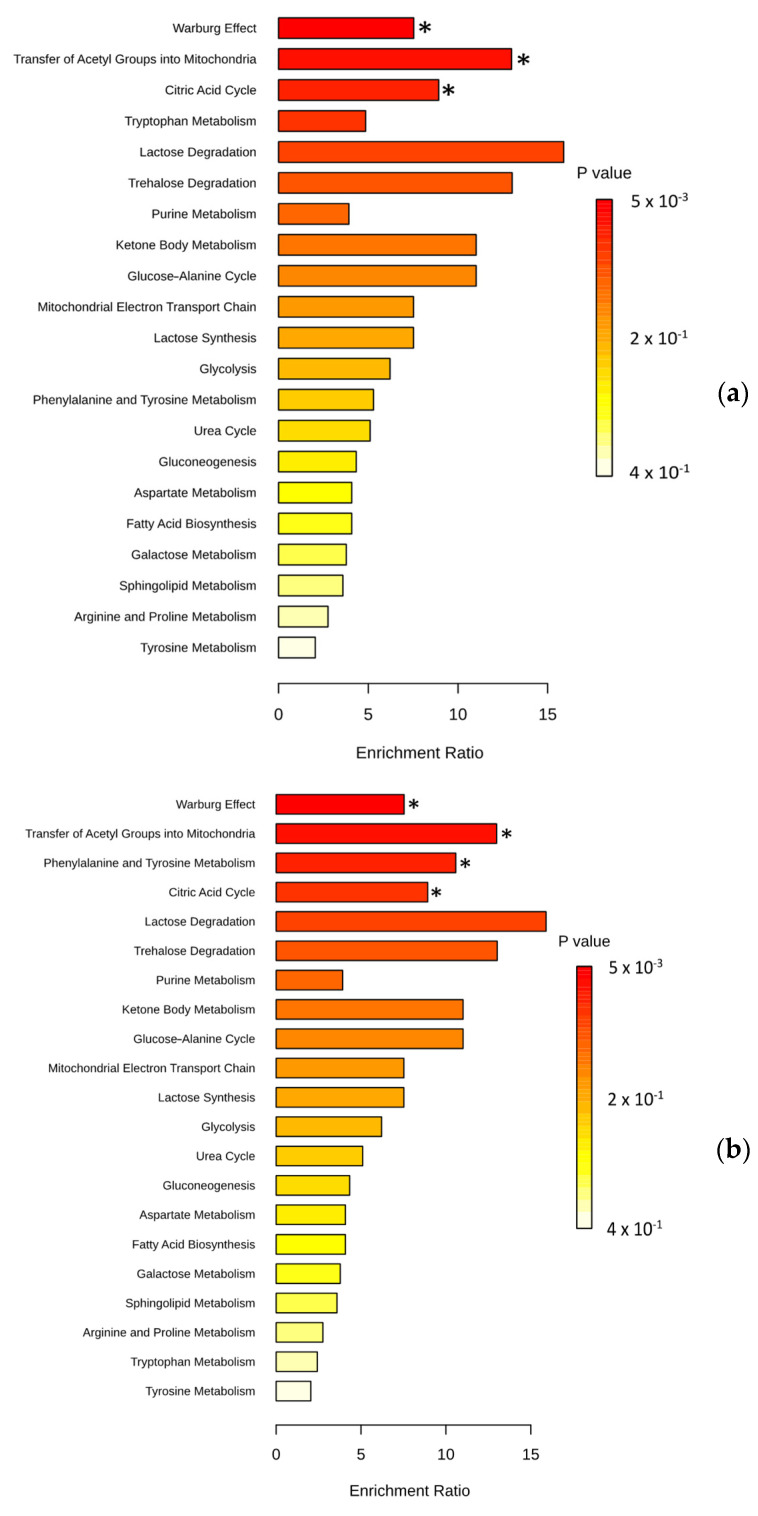

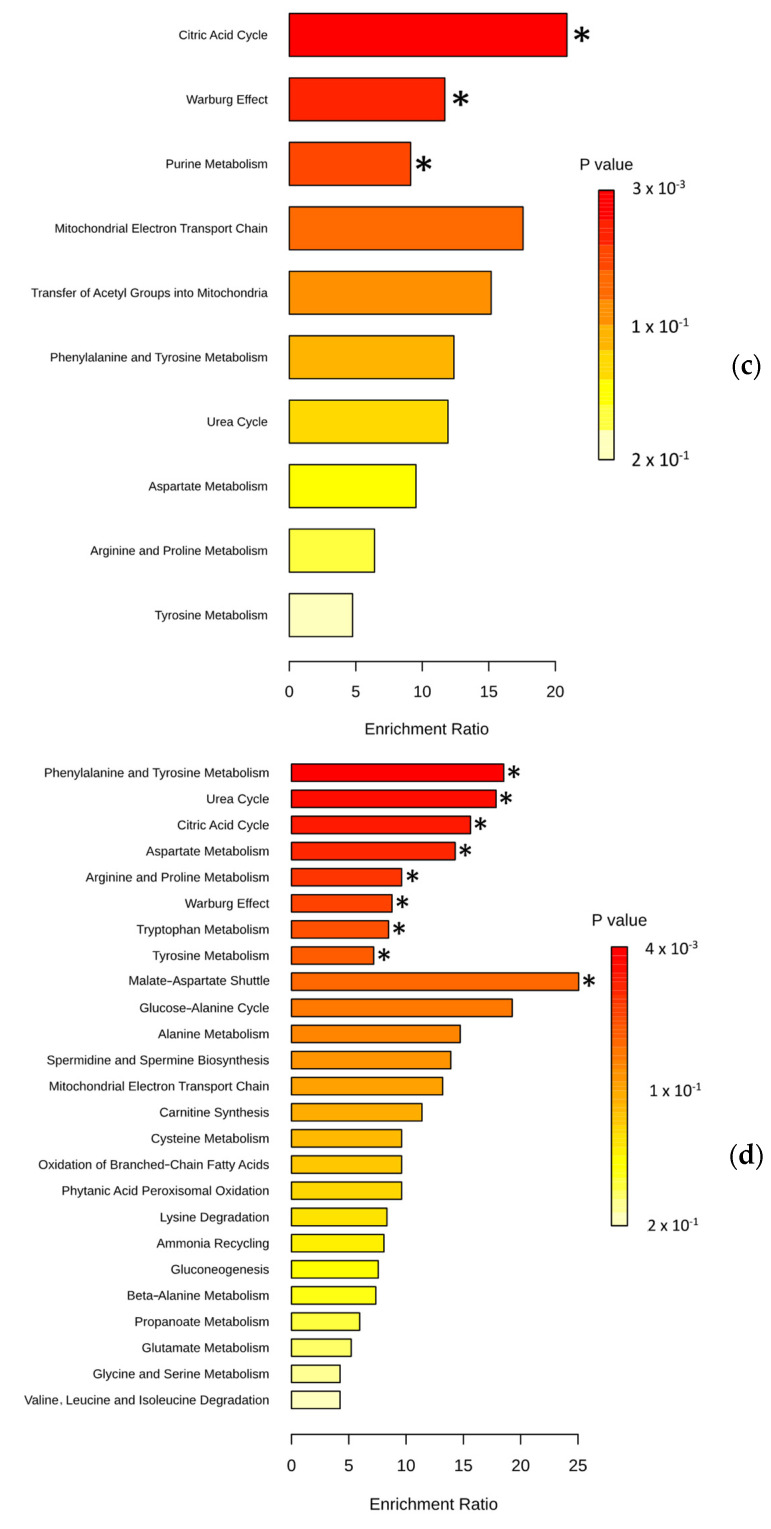
Metabolic pathways expected to be affected by the presence of NENs: (**a**) Enrichment analysis with 9 non-lipid metabolites (among the 21) differentially expressed in patients with NENs compared to healthy people. (**b**) Enrichment analysis with 8 non-lipid metabolites (among 18) differentially expressed in patients with carcinoid tumors compared to healthy people. (**c**) Enrichment analysis with 4 non-lipid metabolites (among 5) differentially expressed in patients with SCLCs compared to healthy people. (**d**) Enrichment analysis with 5 non-lipid metabolites (among 10) differentially expressed in patients with LCNECs compared to healthy people. Representation of the top 25 pathways among the 26 enriched. Set enrichment analysis (MSEA) performed with MetaboAnalyst 6.0. * indicates significantly enriched metabolic pathways (*p* < 0.05).

To understand how changes in plasma metabolite composition affect patients, we focused on identifying which metabolic pathways were impacted. Among the lipids distinguishing NEN patients from healthy individuals, fatty acid biosynthesis (SMP0000456) and beta-oxidation (SMP0000051) were impacted by the presence of each of these cancers. Therefore, we performed an enrichment analysis on the remaining metabolites (non-lipids) (Figure 3, Supplementary Tables S7–S10). A total of 21 metabolic pathways were enriched in the presence of NENs (Figure 3a); among these, only the Warburg effect pathway, the transfer of acetyl groups into mitochondria, and the citric acid cycle were found to be significant (*p* < 0.05) (Supplementary Table S11). In each of the NEN subtypes, different metabolic pathways were significantly impacted. The Warburg effect pathway (*p* = 5.19 × 10^−3^), the transfer of acetyl groups into mitochondria (*p* = 9.05 × 10^−3^), phenylalanine/tyrosine metabolism (*p* = 0.01), and the citric acid cycle (*p* = 0.02) were significantly enriched in the presence of carcinoid tumors (Figure 3b, Supplementary Table S12). Among the significantly enriched pathways in SCLC, purine metabolism (*p* = 0.01) was specific to this subtype (Figure 3c, Supplementary Table S13). In patients with LCNECs, nine metabolic pathways were significantly impacted, six of which were not found in other NENs, including the urea cycle (Figure 3d, Supplementary Table S14).

### 3.4. Plasma Metabolite Profile Can Predict Cancer Subtypes

**Figure 4:**
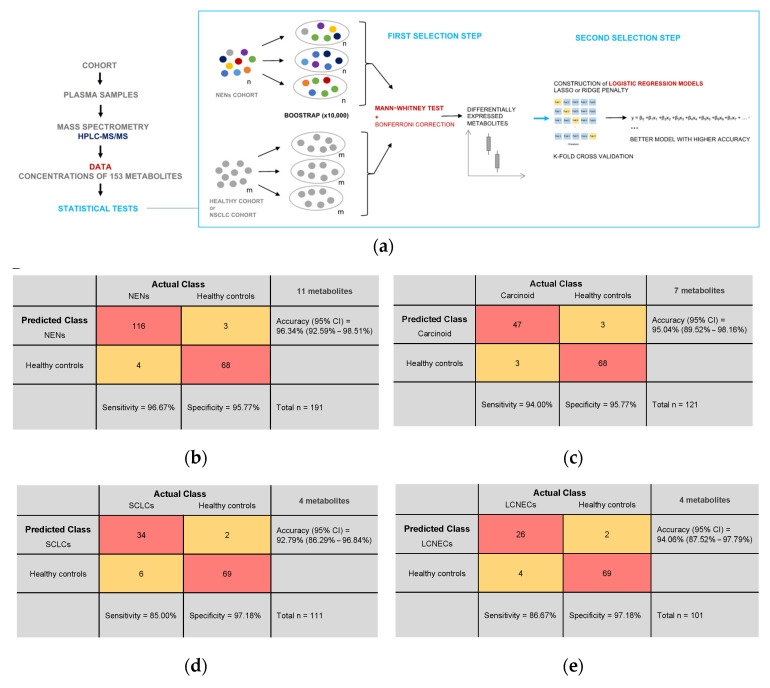
Machine learning with potential plasma metabolite biomarkers: (**a**) Study design. Plasma samples from a total of 657 participants were analyzed by mass spectrometry. Statistical tests using the concentrations of 153 metabolites for each sample were carried out to try and discriminate a particular profile of NENs. First, a selection step based on a Bonferroni-corrected Mann–Whitney test with a bootstrap method identified metabolites differentially expressed in NENs. Finally, among these metabolites, those with predictive potential were used to build logistic regression models. (**b**–**e**) Confusion matrices for all logistic regression models constructed. Test population = entire cohort. (**b**) Prediction of NEN patients and healthy controls using concentrations of the 11 metabolites used in the model. Cross-validation accuracy (95% CI) = 93.16% (87.69%–98.62%); Sensitivity = 95.77%; Specificity = 96.67%. (**c**) Prediction of carcinoid tumor patients and healthy controls using concentrations of the 7 metabolites used in the model. Cross-validation accuracy (95% CI) = 92.60% (84.20%–100%); Sensitivity = 95.78%; Specificity = 94%. (**d**) Prediction of patients with SCLCs and healthy controls using concentrations of the 4 metabolites used in the model. Cross-validation accuracy (95% CI) = 90.08% (82.70%–97.46%); Sensitivity = 97.19%; Specificity = 85%. (**e**) Prediction of LCNEC patients and healthy controls using concentrations of the 4 metabolites used in the model. Cross-validation accuracy = 92.09% (85.06%–99.13%); Sensitivity = 97.19%; Specificity = 86.67%.

Logistic regression models using elastic net regularization were constructed based on a selection of metabolites whose plasma concentrations were significantly altered in patients compared to healthy controls. The final model for distinguishing NEN patients from healthy controls included the following metabolites: 5-hydroxy-indoleacetic acid, C10:1, C10:2, C5DC, fumaric acid, lysoPC a C16:0, lysoPC a C18:0, lysoPC a C18:2, N-acetylputrescine, PC aa C40:2, and uric acid. This model achieved an accuracy of 93.15% ± 5.44% during cross validation, with a sensitivity of 95.77% and a specificity of 96.67%. When tested on the entire cohort of NEN patients and healthy controls, it achieved a classification accuracy of 96.34%, a sensitivity of 96.67%, and a specificity of 95.77% (Figure 4b). To detect carcinoid tumors, a model was constructed using seven metabolites: C10, C18:1, C9, citric acid, fumaric acid, glucose, and phenylalanine. The cross-validation accuracy was 92.60% ± 8.4%, with a sensitivity of 95.78% and a specificity of 94%. When tested, the model’s accuracy reached 95.04%, with a sensitivity of 94% and a specificity of 95.77% (Figure 4c). For distinguishing SCLC patients from healthy individuals, a model was constructed using four metabolites: fumaric acid, lysoPC a C16:0, N-acetylputrescine, uric acid, and C5DC. The cross-validation accuracy was 90.08% ± 7.38%, with a sensitivity of 97.19% and a specificity of 85%. When tested, the model achieved an accuracy of 92.79%, with a sensitivity of 85% and a specificity of 97.18% (Figure 4d). To identify LCNEC patients, a model was built using four metabolites: lysoPC a C16:0, fumaric acid, indolepropionic acid, and C5DC. The cross-validation accuracy was 92.09% ± 7.03%, with a sensitivity of 97.19% and a specificity of 86.67%. When tested on the entire cohort of LCNEC patients and control groups, the accuracy increased to 94.06%, with a sensitivity of 86.67% and a specificity of 97.18% (Figure 4e).

## 4. Discussion

Our study reveals distinct plasmatic metabolic compositions in patients with pulmonary NENs, representing the largest NEN cohort to date. We analyzed 153 general metabolites involved in common cell energy processes, identifying 23 metabolites linked to the presence of NENs. Notably, we found that NEN subtypes displayed distinct plasma metabolic profiles, allowing for the accurate classification (>90%) of NEN subtypes using a machine learning approach.

For the first time, all neuroendocrine tumor subtypes were extensively characterized in blood using metabolomics. We postulate that the metabolomic profile of NENs reflects the deregulation of the general metabolisms of healthy human cells. Our findings underscore the impact of major metabolic pathways in NENs, including lipid metabolism, the tricarboxylic acid cycle, and amino acid metabolism. The identification of several metabolites associated with the Warburg effect suggests that these observations, at least in part, are attributable to tumor cells.

The perturbation observed in lipid metabolism may stem from a shift in the balance between the fatty acid biosynthesis pathway and the beta-oxidation pathway, crucial for meeting cellular energy demands. Cancer cell proliferation heavily relies on lipid metabolism to fuel energy requirements and facilitate the synthesis of membranes and signaling molecules [17,18]. Lipids have previously demonstrated their potential as biomarkers in lung cancer studies [19]. Since its discovery in 1956, the Warburg effect has been associated with cancer cells, characterized by their preference for aerobic glycolysis over oxidative phosphorylation for glucose metabolism [20]. Our enrichment analysis indeed corroborates the presence of this effect across all NEN types (Figure 3b–d).

The involvement of the citric acid cycle in cancer initiation is well documented, owing to mutations in enzymes within this cycle [21]. This could elucidate the tumorigenesis of NENs. Among the identified amino acid metabolisms, tryptophan has been implicated in driving cancer progression [22] and could therefore contribute to the progression of NENs.

Given the heterogeneity of NEN histological subtypes, our findings support the presence of metabolic signatures specific to carcinoid tumors, SCLCs, and LCNECs. We believe that the tumor metabolism of each histological subtype would differ based on general dysregulated metabolisms. Individually, metabolites have the potential to explain tumor behavior and become biomarkers. For example, our results showed hyperglycemia in patients with carcinoid tumors, a phenomenon also observed in many cancers, including NSCLC [23]. This hyperglycemia is not a consequence of tumor progression, but rather a condition favorable to tumorigenesis. The avidity of cancer cells for glucose contributes to their resistance to apoptosis [24]. In addition, we also observed a significant decrease in asymmetric dimethylarginine (ADMA), a product of protein methylation. This metabolite is an inhibitor of nitric oxide synthesis (NOS), and therefore, an inhibitor of angiogenesis [25]. Carcinoid tumors could, therefore, be subject to angiogenesis. This observation is consistent with the characteristics of prominent vascularization and angiogenesis of carcinoid tumors [26].

Purine metabolism exhibited enrichment in SCLCs (Figure 3c). A previous investigation pinpointed dysfunction in this metabolic pathway as a potential driver of tumor progression in ovarian cancer [27]. Hence, it is conceivable that our observed enrichment signifies a mechanism facilitating the advancement of these SCLCs. We observed a decrease in alpha-ketoglutaric acid levels with LCNECs (Figure 1d). This metabolite, also known as 2-oxoglutarate (2OG), serves as a substrate for 2-oxoglutarate-dependent dioxygenase (2OGDD) enzymes involved in cancer metabolism and epigenetics. While some 2OGDDs promote tumor growth and others suppress it, the precise mechanisms remain unclear [28]. As LCNECs are rapidly growing tumors, it is possible that a decrease in 2OG limits 2OGDD activity, which suppresses tumor growth. Thus, we believe that the reduction of 2OG in LCNECs might contribute to tumorigenesis. The significant decrease in 2OG levels in LCNECs compared to healthy controls and NSCLCs underscores its potential as a plasma biomarker. In this study, a decreased plasma concentration of indolepropionic acid (IPA) was specifically observed in patients with LCNECs. IPA, generated by the human microbiota, is recognized for its anticancer properties. Previous research by Sàri et al. demonstrated that IPA has the ability to diminish the proportions of cancer stem cells and inhibit their proliferation in cellular and animal models [29]. The reduced plasma concentration of IPA in LCNEC patients suggests a potential limitation of its anticancer effects, possibly contributing to the pathogenesis of this cancer type. Finally, a notable increase in spermine levels was observed in LCNECs (Figure 1d). This polyamine has previously been associated with promoting an immunosuppressive environment within the tumor microenvironment [30]. We speculate that spermine contributes to establishing an immunosuppressive tumor environment for LCNECs.

We attempted to detect the smallest differences between SCLCs and LCNECs by conducting a comparative analysis; however, none of the 153 candidate metabolites could discriminate between the two subtypes, which may be due to the fact that these two subtypes share a similar metabolomic profile given their similarity in terms of pathology and genomic features [31,32]. In addition, NECs also share histological characteristics with NSCLCs such as adenocarcinomas and squamous cell carcinomas [33,34], with up to 25% of LCNECs and SCLCs harboring an NSCLC component upon resection [1]. Eleven out of the 70 NECs (16%) included in our study indeed presented with a minor NSCLC component. Our comparison of the metabolism between NECs and NSCLCs identified only five metabolites with significantly divergent concentrations. Our findings suggest that the metabolism of NECs and NSCLCs most likely overlaps. Nonetheless, future investigations employing a broader array of metabolites could provide further insights to corroborate or refute this observation.

The use of machine learning with metabolomics for cancer diagnosis is now expanding rapidly [35]. In our study, regularization regression models were used to classify NEN subtypes, including carcinoid tumors, SCLCs, LCNECs, and healthy controls, based on identified metabolites. Although we did not have access to an external validation cohort, we opted for an experimental design capable of estimating performance parameters by using cross validation (Figure 4a). This strategy enabled us to consistently assess the classification accuracy of each patient, even for the smallest cohort (LCNECs, *n* = 30). Unsupervised clustering revealed two primary groups: one encompassing healthy individuals and some carcinoid tumors, and the other including the remaining carcinoid tumors, SCLCs, and LCNECs (Figure 2b). Notably, clinical features did not cluster to reflect tumor and control categories.

The relationship between metabolites and NENs extends beyond pulmonary NENs, reflecting a complex interplay of altered metabolic pathways in these tumors. Metabolomics has emerged as a crucial tool for understanding NENs, as these tumors, like many cancers, undergo significant metabolic reprogramming to sustain rapid growth and adapt to various environmental conditions. This metabolic reprogramming, as previously discussed, commonly involves alterations in glucose, amino acid, and lipid metabolism. For example, metabolomic studies of small intestinal NENs have identified specific metabolite profiles, such as elevated levels of tryptophan, serotonin, and related metabolites, which correlate with the hormonal activity of these tumors [36]. In pheochromocytomas, a type of NEN originating from the adrenal medulla, distinctive patterns of catecholamine metabolism, including elevated levels of normetanephrine and metanephrine, have proven useful for diagnosis and monitoring [37]. Furthermore, the development of a class prediction model utilizing peptides generated by MALDI mass spectrometry imaging has facilitated the differentiation of pancreatic ductal adenocarcinoma from pancreatic neuroendocrine tumors, underscoring the potential of peptide-based metabolomic approaches in tumor classification [38]. These advancements highlight the significant role of metabolomics in elucidating metabolic alterations in NENs and improving diagnostic and monitoring strategies.

Our findings advance our understanding of cancer metabolomics and hold promise for clinical applications in diagnosis, monitoring, and prognosis. For pulmonary NENs, measuring specific metabolites would be critical across various stages of patient management, including diagnosis, subtyping, monitoring treatment response, and detecting recurrence. Clinicians should tailor these measurements to the specific clinical context, ensuring that the appropriate biomarkers are assessed to guide diagnosis and treatment. These metabolites would be particularly valuable for the early diagnosis of NENs, allowing for timely intervention. Furthermore, regular monitoring during treatment can help assess therapeutic response and detect early signs of disease progression or recurrence. In the era of precision oncology, these results underscore the significance of targeting cancer metabolism, with several anti-metabolite drugs in clinical use, especially those targeting nucleotide metabolism [39]. Our enrichment analysis (MetaboAnalyst) has demonstrated its usefulness in deciphering the pathways potentially impacted by the presence of NENs. Although strict identification would require in vivo or in vitro experiments, the pathways listed are consistent with what is known of cancer metabolism, such as the Warburg effect found in each tumor subtype (Figure 3). Finally, the metabolites identified in our study need to be confirmed in a larger study. A biomarker test should also use several cohorts, preferably large [40]. Thus, an external validation cohort is required to confirm these results.

## 5. Conclusions

Our study revealed distinct metabolic differences in the blood of NEN patients for the first time. This research contributes to the field of cancer metabolomics, specifically for NENs, and offers potential benefits for detection, diagnosis, and predictive biomarker development.

## Figures and Tables

**Table 1:** Characteristics of NEN patients and controls.

Characteristics	Cases (*n* = 120)	Controls (*n* = 71)	*p*-Value
Age (years), mean ± SD	61.9 ± 9.8	56.7 ± 10.7	0.16
Carcinoids (*n* = 50)	59.3 ± 11.2		0.89
SCLC (*n* = 40)	63.3 ± 8.1		4.73 × 10^−2^
LCNEC (*n* = 30)	64.1 ± 8.9		0.03
Sex (%)			0.29
Male	36.7	45.1	
Female	63.3	54.9	
Smoking status (%)			1.09 × 10^−7^
Current smokers	23.3	8.4	
Ex-smokers	58.3	35.2	
Non-smokers	18.3	56.3	
BMI (kg/m^2^), mean ± SD	27.8 ± 4.7	26.7 ± 6.0	0.19

BMI, body mass index; SD, standard deviation.

**Table 2:** Characteristics of NENs patients compared to NSCLC patients.

Characteristics	Cases (*n* = 120)	NSCLC (*n* = 466)	*p*-Value
Age (years), mean ± SD	61.9 ± 9.8	65.2 ± 8.1	6.50 × 10^−4^
Carcinoids (*n* = 50)	59.3 ± 11.2		
SCLC (*n* = 40)	63.3 ± 8.1		
LCNEC (*n* = 30)	64.1 ± 8.9		
Sex (%)			7.70 × 10^−2^
Male	36.7	50.1	
Female	63.3	49.9	
Smoking status (%)			1.80 × 10^−2^
Current smokers	23.3	22.8	
Ex-smokers	58.3	72.2	
Non-smokers	18.3	4.6	
Passive	0	0.4	
BMI (kg/m^2^), mean ± SD	27.8 ± 4.7	27.1 ± 5.1	0.24

BMI, body mass index; SD, standard deviation.

## Data Availability

The data presented here are available from the corresponding author Philippe Joubert.

## Supplementary Materials

The following supporting information can be downloaded at: https://www.mdpi.com/article/10.3390/cancers16183179/s1. Table S1: Twenty-one metabolites significantly discriminating between NEN patients and healthy people; Table S2: Eighteen metabolites significantly discriminating between carcinoid tumor patients and healthy people; Table S3: Five metabolites significantly discriminating between SCLC patients and healthy people; Table S4: Ten metabolites significantly discriminating between LCNEC patients and healthy people; Table S5: Four metabolites significantly discriminating between SCLC patients and NSCLC patients; Table S6: Two metabolites significantly discriminating between LCNEC patients and NSCLC patients; Table S7: Nine metabolites significantly discriminating between NEN patients and healthy people used for enrichment analysis; Table S8: Eight metabolites significantly discriminating between carcinoid tumor patients and healthy people used for enrichment analysis; Table S9: Four metabolites significantly discriminating between SCLC patients and healthy people used for enrichment analysis; Table S10: Five metabolites significantly discriminating between LCNEC patients and healthy people used for enrichment analysis; Table S11: Metabolic pathways significantly identified after MSEA in NENs patients; Table S12: Metabolic pathways significantly identified after MSEA in carcinoid tumor patients; Table S13: Metabolic pathways significantly identified after MSEA in SCLC patients; Table S14: Metabolic pathways significantly identified after MSEA in LCNEC patients.
