# Supplementary material for: Metabolomic Profiling of Pulmonary Neuroendocrine Neoplasms
Source: Cancers (Basel). 2024 Sep 17;16(18):3179. doi: 10.3390/cancers16183179 (PMC11429548; doi:10.3390/cancers16183179)
Supplement: Supplementary file 1 [file cancers-16-03179-s001.zip › cancers-3174561-supplementary final 1.pdf]

Table S1. Twenty-one metabolites significantly discriminating between NENs patients and healthy people. Bonferronicorrected Mann-Whitney test on metabolite concentrations normalized by Z-score.

Metabolite name	Median P-value	Fold Change
5-Hydroxy Indoleacetic acid	2.42e-4	1.19
beta-Hydroxybutyric acid	1.02e-5	2.48
C10	4.19e-5	0.71
C10:1	2.02e-4	0.86
C10:2	1.67e-5	1.30
C5	5.84e-5	0.83
C5DC	7.89e-8	1.20
Citric acid	2.22e-9	0.74
Fumaric acid	5.46e-14	0.57
Glucose	1.21e-4	1.16
Indolepropionic acid	6.88e-5	0.65
LysoPC a C16:0	3.08e-11	0.72
LysoPC a C16:1	4.12e-5	0.79
LysoPC a C17:0	2.91e-7	0.73
LysoPC a C18:0	1.30e-6	0.78
LysoPC a C18:2	9.97e-7	0.68
LysoPC a C24:0	6.71e-5	0.86
N-acetylputrescine	1.42e-4	1.19
PC aa C40:2	1.66e-6	0.79
Tryptophan	2.91e-7	0.78
Uric acid	8.75e-6	0.79

Table S2. Eighteen metabolites significantly discriminating between carcinoid tumors patients and healthy people. Bonferroni-corrected Mann-Whitney test on metabolite concentrations normalized by Z-score.

Metabolite name	Median P-value	Fold Change
Asymmetric dimethylarginine	1.64e-4	0.86
beta-Hydroxybutyric acid	9.92e-5	2.90
C10	1.28e-4	0.70
C18:1	2.48e-4	1.24
C5DC	5.44e-6	1.19
C9	1.35e-4	1.20
Citric acid	4.36e-8	0.73
Fumaric acid	2.31e-9	0.60
Glucose	1.00e-5	1.20
LysoPC a C16:0	3.54e-8	0.73
LysoPC a C16:1	4.15e-5	0.76
LysoPC a C17:0	9.64e-7	0.70
LysoPC a C18:0	4.03e-5	0.79
LysoPC a C18:2	3.91e-5	0.69
PC aa C40:2	3.31e-6	0.78
Phenylalanine	9.24e-5	0.87
Tryptophan	5.99e-6	0.77
Uric acid	3.23e-4	0.81

Table S3. Five metabolites significantly discriminating between SCLCs patients and healthy people. Bonferroni-corrected Mann-Whitney test on metabolite concentrations normalized by Zscore.

Metabolite name	Median P-value	Fold Change
Citric acid	3.17e-6	0.73
Fumaric acid	4.59e-9	0.54
LysoPC a C16:0	2.84e-6	0.74
N-acetylputrescine	1.38e-4	1.26
Uric acid	3.24e-4	0.77

Table S4. Ten metabolites significantly discriminating between LCNECs patients and healthy people. Bonferroni-corrected Mann-Whitney test on metabolite concentrations normalized by Z-score.

Metabolite name	Median P-value	Fold Change
alpha-Ketoglutaric acid	9.16e-5	0.76
C5DC	4.90e-5	1.26
Fumaric acid	3.49e-7	0.57
Indolepropionic acid	3.06e-6	0.34
LysoPC a C16:0	1.72e-6	0.66
LysoPC a C18:0	1.67e-4	0.70
LysoPC a C18:2	3.16e-4	0.62
PC aa C36:6	2.48e-6	0.53
Spermine	2.30e-4	1.16
Tryptophan	2.24e-4	0.74

Table S5. Four metabolites significantly discriminating between SCLCs patients and NSCLCs patients. Bonferroni-corrected Mann-Whitney test on metabolite concentrations normalized by Z-score.

Metabolite name	Median P-value	Fold Change
5-Hydroxy Indoleacetic acid	4.87e-5	1.38
C12:1	1.03e-4	0.79
Fumaric acid	1.91e-6	0.57
Succinic acid	9.69e-5	1.33

Table S6. Two metabolites significantly discriminating between LCNECs patients and NSCLCs patients. Bonferroni-corrected MannWhitney test on metabolite concentrations normalized by Z-score.

Metabolite name	Median P-value	Fold Change
alpha-Ketoglutaric acid	2.52e-4	0.75
Fumaric acid	3.82e-5	0.60

Table S7. Nine metabolites significantly discriminating between NENs patients and healthy people used for enrichment analysis. MetaboAnalyst.

Query	Hit	HMDB	PubChem	KEGG
5-Hydroxy Indoleacetic acid	5-Hydroxyindoleacetic acid	HMDB0000763	1826	C05635
beta-Hydroxybutyric acid	3-Hydroxybutyric acid	HMDB0000011	92135	C01089
Citric acid	Citric acid	HMDB0000094	311	C00158
Fumaric acid	Fumaric acid	HMDB0000134	444972	C00122
Glucose	D-Glucose	HMDB0000122	64689	C00221
Indolepropionic acid	Indole-3-propionic acid	HMDB0002302	3744	NA
N-acetylputrescine	N-Acetylputrescine	HMDB0002064	122356	C02714
Tryptophan	L-Tryptophan	HMDB0000929	6305	C00078
Uric acid	Uric acid	HMDB0000289	1175	C00366

Table S8. Eight metabolites significantly discriminating between carcinoid tumors patients and healthy people used for enrichment analysis. MetaboAnalyst.

Query	Hit	HMDB	PubChem	KEGG
Asymmetric dimethylarginine	Asymmetric dimethylarginine	HMDB0001539	123831	C03626
beta-Hydroxybutyric acid	3-Hydroxybutyric acid	HMDB0000011	92135	C01089
Citric acid	Citric acid	HMDB0000094	311	C00158
Fumaric acid	Fumaric acid	HMDB0000134	444972	C00122
Glucose	D-Glucose	HMDB0000122	64689	C00221
Phenylalanine	Phenylalanine	HMDB0000159	6140	C00079
Tryptophan	L-Tryptophan	HMDB0000929	6305	C00078
Uric acid	Uric acid	HMDB0000289	1175	C00366

Table S9. Four metabolites significantly discriminating between SCLCs patients and healthy people used for enrichment analysis. MetaboAnalyst.

Query	Hit	HMDB	PubChem	KEGG
Citric acid	Citric acid	HMDB0000094	311	C00158
Fumaric acid	Fumaric acid	HMDB0000134	444972	C00122
N-acetylputrescine	N-Acetylputrescine	HMDB0002064	122356	C02714
Uric acid	Uric acid	HMDB0000289	1175	C00366

Table S10. Five metabolites significantly discriminating between LCNECs patients and healthy people used for enrichment analysis. MetaboAnalyst.

Query	Hit	HMDB	PubChem	KEGG
alpha-Ketoglutaric acid	Oxoglutaric acid	HMDB0000208	51	C00026
Fumaric acid	Fumaric acid	HMDB0000134	444972	C00122
Indolepropionic acid	Indole-3-propionic acid	HMDB0002302	3744	NA
Spermine	Spermine	HMDB0001256	1103	C00750
Tryptophan	L-Tryptophan	HMDB0000929	6305	C00078

Table S11. Metabolic pathways significantly identified after MSEA in NENs patients. MetaboAnalyst 6.0.

Metabolite Set	Metabolite hits	P-value	Holm	FDR
Warburg Effect	Citric acid, D-Glucose, Fumaric acid	5.19e-3	0.51	0.44
Transfer of Acetyl Groups into Mitochondria	Citric acid, D-Glucose	9.05e-3	0.88	0.44
Citric Acid Cycle	Citric acid, Fumaric acid	0.02	1	0.61

Table S12. Metabolic pathways significantly identified after MSEA in carcinoid tumors patients. MetaboAnalyst 6.0.

Metabolite Set	Metabolite hits	P-value	Holm	FDR
Warburg Effect	Citric acid, D-Glucose, Fumaric acid	5.19e-3	0.51	0.44
Transfer of Acetyl Groups into Mitochondria	Citric acid, D-Glucose	9.05e-3	0.88	0.44
Phenylalanine and Tyrosine Metabolism	Fumaric acid, Phenylalanine	0.01	1	0.44
Citric Acid Cycle	Citric acid, Fumaric acid	0.02	1	0.46

Table S13. Metabolic pathways significantly identified after MSEA in SCLCs patients. MetaboAnalyst 6.0.

Metabolite Set	Metabolite hits	P-value	Holm	FDR
Citric Acid Cycle	Citric acid, Fumaric acid	2.91e-3	0.28	0.28
Warburg Effect	Citric acid, Fumaric acid	9.20e-3	0.89	0.45
Purine Metabolism	Fumaric acid, Uric acid	0.01	1	0.49

Table S14. Metabolic pathways significantly identified after MSEA in LCNECs patients. MetaboAnalyst 6.0.

Metabolite Set	Metabolite hits	P value	Holm P	FDR
Phenylalanine and Tyrosine Metabolism	Fumaric acid, Oxoglutaric acid	4.06e-3	0.40	0.17
Urea Cycle	Fumaric acid, Oxoglutaric acid	4.37e-3	0.42	0.17
Citric Acid Cycle	Fumaric acid, Oxoglutaric acid	5.70e-3	0.55	0.17
Aspartate Metabolism	Fumaric acid, Oxoglutaric acid	6.81e-3	0.65	0.17
Arginine and Proline Metabolism	Fumaric acid, Oxoglutaric acid	0.01	1	0.26
Warburg Effect	Fumaric acid, Oxoglutaric acid	0.02	1	0.26
Tryptophan Metabolism	Oxoglutaric acid, L-Tryptophan	0.02	1	0.26
Tyrosine Metabolism	Fumaric acid, Oxoglutaric acid	0.03	1	0.32
Malate-Aspartate Shuttle	Oxoglutaric acid	0.04	1	0.43
